# A Randomized Controlled Phase IIb Trial of Antigen-Antibody Immunogenic Complex Therapeutic Vaccine in Chronic Hepatitis B Patients

**DOI:** 10.1371/journal.pone.0002565

**Published:** 2008-07-02

**Authors:** Dao-Zhen Xu, Kai Zhao, Li-Min Guo, Xin-Yue Chen, Hui-Fen Wang, Ji-Ming Zhang, Qin Xie, Hong Ren, Wen-Xiang Wang, Lan-Juan Li, Min Xu, Pei Liu, Jun-Qi Niu, Xue-Fan Bai, Xin-Liang Shen, Zheng-Hong Yuan, Xuan-Yi Wang, Yu-Mei Wen

**Affiliations:** 1 Ditan Hospital, Beijing, China; 2 Beijing Institute of Vaccine and Biological Products, Beijing, China; 3 You-An Hospital, Capital Medical University, Beijing, China; 4 302 Military Hospital, Beijing, China; 5 Hua-Shan Hospital, Fudan University, Shanghai, China; 6 Rui-Jin Hospital, Shanghai Jiaotong University, Shanghai, China; 7 Second affiliated hospital, Chongqing Medical University, Chongqing, China; 8 First affiliated hospital, Chongqing Medical University, Chongqing, China; 9 First affiliated hospital, Zhejiang University, Hanzhou, Zhejiang Province, China; 10 Guangzhou Eighth Hospital, Guangzhou, Guangdong Province, China; 11 Second Hospital Affiliated to China Medical University, Shenyang, Liaoning Province, China; 12 First affiliated hospital, Jilin University, Changchun, Jilin Province, China; 13 Tangdu Hospital, Fourth Military Medical University, Xi'an, Shanxi Province, China; 14 Key Laboratory Medical Molecular Virology, Shanghai Medical College, Fudan University, Shanghai, China; 15 Institute of Biological Sciences, Fudan University, Shanghai, China; Karolinska Institutet, Sweden

## Abstract

**Background:**

The safety of the immune complexes composed of yeast-derived hepatitis B surface antigen (HBsAg) and antibodies (abbreviated as YIC) among healthy adults and chronic hepatitis B patients has been proved in phase I and phase IIa trial. A larger number of patients for study of dosage and efficacy are therefore needed.

**Methods and Principal Findings:**

Two hundred forty two HBeAg-positive chronic hepatitis B patients were immunized with six injections of either 30 µg YIC, 60 µg of YIC or alum adjuvant as placebo at four-week intervals under code. HBV markers and HBV DNA were monitored during immunization and 24 weeks after the completion of immunization. The primary endpoint was defined as loss of HBeAg, or presence of anti-HBe antibody or suppression of HBV DNA, while the secondary endpoint was both HBeAg seroconversion and suppression of HBV DNA. Statistical significance was not reached in primary endpoints four weeks after the end of treatment among three groups, however, at the end of follow-up, HBeAg sero-conversion rate was 21.8%(17/78) and 9% (7/78) in the 60 µg YIC and placebo groups respectively (p = 0.03), with 95% confidence intervals at 1.5% to 24.1%. Using generalized estimating equations (GEEs) model, a significant difference of group effects was found between 60 µg YIC and the placebo groups in terms of the primary endpoint. Eleven serious adverse events occurred, which were 5.1%, 3.6%, and 5.0% in the placebo, 30 µg YIC and 60 µg YIC groups respectively (p>0.05).

**Conclusions:**

Though statistical differences in the preset primary and secondary endpoints among the three groups were not reached, a late and promising HBeAg seroconversion effect was shown in the 60 µg YIC immunized regimen. By increasing the number of patients and injections, the therapeutic efficacy of YIC in chronic hepatitis B patients will be further evaluated.

**Trial Registration:**

ChiCTR.org ChiCTR-TRC-00000022

## Introduction

According to the World Health Organization, there are 350 million people worldwide, who are chronically infected with HBV. Prolonged chronic hepatitis B results in the development of liver cirrhosis, liver failure, or hepatocellular carcinoma[1]. The pathogenesis of HBV in chronically infected patients has been well- studied and reviewed. Lack of effective immune responses, notably, defective cell-mediated immune responses (CD4, CD8 and NK cells, cytolytic responses) against HBV, defective dendritic cell (DC) functions and imbalance of cytokine production have been identified as the major mechanisms for virus persistence and initiation of chronic liver disease [2], [3], [4], [5], [6]. Effective host immune responses are crucial to terminate viral persistence. To overcome the defects in immune responses, various therapeutic measures have been designed to boost effective host immune responses [7], [8], [9], [10], [11], [12], [13]. Immune complexes (IC) composed of antigen and antibodies have long been used to induce potent antibody responses against microbial proteins and other proteins in animals [14]. Whether IC can be used for therapeutic treatment of viral hepatitis B patients has been questioned because circulating immune complexes (CIC) have been found in some chronic hepatitis B patients [15]. We hypothesized that the crucial difference between CIC and the immune complexes composed of yeast-derived hepatitis B surface antigen (HBsAg) and antibodies (abbreviated as YIC) used in this study is that, in CIC, the anti-HBs antibodies from the patient are of low affinity, which cannot efficiently bind to HBsAg and clear the protein from the host. In contrast, the anti-HBs used to produce YIC are generated from healthy adults who were immunized multiple times with yeast-derived recombinant HBsAg. Therefore, these are high affinity antibodies that can combine efficiently with HBsAg [16]. When YIC is administered via intramuscular injections, it served as an immunogen to the host, and antigen presenting cells in the immune tolerant host would be forced to uptake the HBsAg complexed to its antibodies via the Fc receptors on antigen presenting cells, and thereby leading to modified antigen processing and presentation in the complex. This hypothesis has been confirmed by our previous experimental studies in animal models and *in vitro* experiments on human dendritic cells [17], [18]. A recent preliminary study in a small number of chronic hepatitis B patients showed that the therapeutic effect of YIC correlated with both cytolytic and noncytolytic responses [19].Though antiviral drugs are highly effective in inhibiting HBV replication, emergence of drug resistance and rebound of virus replication after withdrawal of drugs are major disadvantages for treatment of persistent viral infections [20], [21]. Conversely, vaccine therapy is an inexpensive and promising approach for the treatment of persistent viral infections [22], [23].

To study the in vivo immunotherapeutic effects of YIC in chronic hepatitis B patients, a double-blind, randomized, placebo-controlled clinical study was conducted, and results are presented.

## Methods

The protocol for this trial and supporting CONSORT checklist are available as supporting information; see Checklist S1 and Protocol S1.

### Immune complexes and placebo

Both the immune complexes and placebo used in this study were manufactured by Beijing Institute of Vaccine and Biological Products, and the Chinese Good manufacture practice (GMP) regulation was followed. Each dose of 1 mL immune complexes (YIC) consisted of either 30 or 60 µg of HBsAg complexed to human anti-HBs immunoglobulin (HBIG) at an appropriate ratio (described in US patent 6,221,664 B1 and European patent 913157), using alum as the adjuvant, which was a mixture of KAl(SO_4_)_2_ and NaOH. The placebo contained 0.1% alum identical to that being used in YIC as the adjuvant.

### Study design

This double-blind, randomized, and placebo-controlled study was conducted at 12 evaluation centers for the treatment of HBV, which were certificated by the State Food and Drug Administration (SFDA), China. Prior to initiating the clinical trial, the protocol of this study was submitted, registered, licensed and approved by the SFDA, China (license number 2002L0038). The trial was registered at WHO International Clinical Trials Registry Platform. Final approval from the ethics committee at Ditan hospital after ethical evaluating at each participating center was completed in February 2005, and enrollment of patients was initialed in March, 2005. Prior to enrollment, each patient signed a written consent for participating in this trial.

The study was designed in a three-arm fashion. Eligible patients were assigned to receive 30 µg YIC, 60 µg YIC or placebo in blocks of 6 (two for the 30 µg YIC vaccine, two for the 60 µg YIC vaccine, and two for the placebo group) through computer generated random numbers on the label of study agent vial in terms of recruiting sequence. An independent biostatistician was in charge of the processing of randomization using SAS program (SAS Institute Inc., Cary, NC, USA). All participants were immunized with six intramuscular injections at 4- week intervals, and followed for 24 weeks after the termination of immunization. Serum samples were collected from each patient at baseline, 12th, 24th, 36th and 44th week after initial injection, and separated into two vials. One vial of serum was used for routine biochemical and hematological tests, such as ALT, AST, and was conducted immediately at each evaluation center. Another vial serum was storied at −70°C at each center and shipped to the reference lab at Beijing Ditan Hospital at the end of study for assays of HBV markers and virus load of all samples from all centers. The study was designed by a chief clinical investigator in Ditan Hospital, and was monitored by TigerMed, China, an independent Contract Research Organization. The principles of good clinical practice and clinical trial-related guidelines issued by SFDA were implemented throughout the study.

### Patients

Patients with chronic hepatitis B, aged 18–65 years old, who were HBsAg and HBeAg positive for at least 6 months and who were anti-HBe negative with an HBV viral load >100,000 copies/mL and a serum ALT of two to ten times the upper limit of normal value within four weeks before randomization were recruited at each evaluation center. Exclusion criteria were co-infection with hepatitis A, C, D and E virus, or HIV; taking antiviral, hepatotoxic or immunosuppressive drugs or products within the preceding 6 months; other causes of liver disease; serious medical or psychiatric illness; hepatic cirrhosis or AFP >100 ng/mL; abnormal serum creatinine, thrombocyte count, hemaglobin or serum total bilirubin; and pregnancy.

### Assays

Routine biochemical and hematological tests, such as ALT, AST, were carried out at each evaluation center using automated techniques available at each center.

At the end of study, all frozen serum samples from all enrolled patients under code were transferred from all evaluation centers to the reference lab at Beijing Ditan Hospital for assays of HBeAg, anti-HBe and serum HBV DNA levels. Samples at all time points were thawed and assayed using the same lot of reagents. Sequential samples from one patient were tested on the same day. Abbott EIA AxSYM (Abbott, Abbott Park, IL, USA) was employed for HBsAg, HBeAg, and anti-HBe. According to protocols provided by the manufacturer, positive and negative cutoffs were calculated, with the positive and negative controls as required by the diagnostic kits. Serum HBV DNA was quantified by fluorescent PCR assay using the ABI equipment, and reagents were from PiJi, Shenzhen Co, China, with a detection limit of 500 copies/mL.

### Endpoints

The virologic response was assessed four weeks after the end of treatment (week 24) and 24 weeks after the end of treatment (week 44, the end of follow-up). HBeAg seroconversion was defined by the loss of HBeAg and the presence of anti-HBe antibody. Suppression of HBV DNA was defined as the >2 log10 decrease of viral load. The primary endpoint was defined as loss of HBeAg, or presence of anti-HBe antibody or suppression of HBV DNA. The secondary endpoint was designated as both HBeAg seroconversion and suppression of HBV DNA.

### Safety

All participants were observed for local reactions and systemic symptoms through diary card and follow-up interview. The causality of adverse events was determined by the clinical investigators, and the criteria for severe adverse events were: blood total bilirubin (TB) >3×17.2 µmol/L), prolonged prothrombin time (PTA) <40%), and ALT levels elevated 10 times higher than that of the baseline. The severity of adverse events was classified as mild (easily tolerated; causing minimal discomfort; not interfering normal everyday activities), moderate (Sufficiently discomforting to interfere with normal everyday activities) and severe (Prevents normal everyday activities). Safety analysis was performed on all patients who underwent randomization and received at least one dose of study agent.

### Data management and statistical analysis

The sample size was calculated to ensure an adequate evaluation of the primary endpoint. Based on the literatures and the results of phase IIa trial, a sample size of 78 patients per arm could detect a difference of primary response rate between 60 µg group, 30 µg group and placebo group (response rate in 60 µg YIC group vs placebo group, 35% vs 3%; and response rate in 30 µg YIC group vs placebo group, 20% vs 3%) with a statistical power of 80% at the 0.05 level of significance, allowing for a dropout rate of 20%.

All data were double entered into custom-made data entry programs. The data management programs included range and consistency checks. An SAS program (SAS Institute Inc., Cary, NC, USA) was applied for statistical analysis. Analysis was conducted on all eligible patients according to the intent-to-treat principle. HBV DNA was logarithmically transformed for analysis. For binary data, the Chi square test, or Fisher's exact test when data were sparse, were employed. For dichotomous outcomes, ANOVA was used.

Repeated measures analysis was performed using a generalized estimating equations (GEEs) method to adjust the dependence among repeated observations made on the same patient while testing the group and time effects [24]. In the model, we included the time effect as a class variable which used three indicator variables. The indicator variable was defined by treating Week 44 as a baseline time. Similarly, the group effect was defined by using two indicator variables, where the placebo group served as baseline group. Since there were only four repeated measurements (week 12, 24, 36, and 44), we applied the unstructured (UN) working covariance matrix which provided robust estimation of covariance to the structure. Since the ALT and HBV DNA are crucial indicators for baseline assessment, adjustments were made for group, baseline ALT and HBV DNA. A p-value <0.05 (two-tailed) was considered statistically significant.

## Results

### Baseline characteristics of enrolled patients

**Figure 1:**
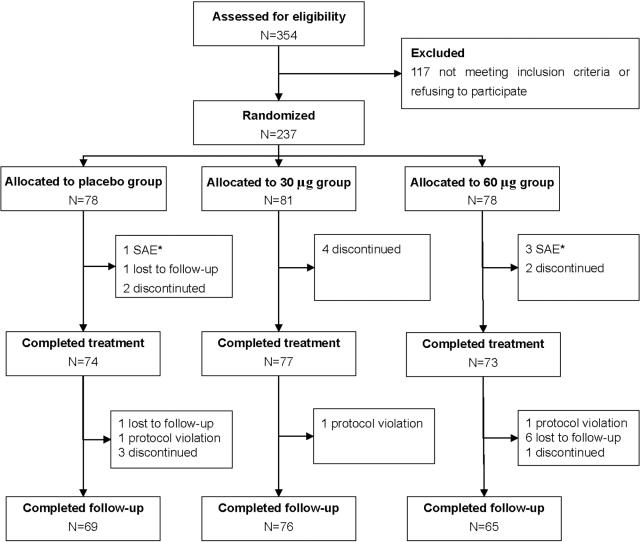
Summary of participants. Footnote: The numbers of SAE patients are those who discontinued treatment, while other SAE patients are not included in this figure.

Three hundred and fifty four hepatitis B patients were evaluated for the inclusion criteria. Of these, 242 patients were eligible and assigned randomly to placebo, 30 µg YIC and 60 µg YIC groups in a three-month period. Five patients were found ineligible during the batch assay at the end of treatment (week 24) at the central laboratory, and therefore were excluded from the intent-to-treat analysis. Of 237 patients included in the analysis , 8 receiving placebo, 5 receiving 30ug YIC, and 10 receiving 60ug YIC either did not complete the treatment, did not complete the follow-up or violated the protocol (Figure 1). No significant difference was found in baseline characteristics among three groups (Table 1).

**Table 1:** Characteristics of participants.

Characteristics	Placebo group (N = 78)	30 µg YIC group (N = 81)	60 µg YIC group (N = 78)
Age (yr; SD)	27.9±7.6	28.6±8.6	28.8±8.0
Female sex (no. ; %)	19 (24.4)	22 (27.2)	17 (21.8)
Weight (kg; SD)	61.7±11.1	62.3±10.0	63.7±9.3
Course of illness, hepatitis B (yr; SD)	6.3±5.2	6.0±5.8	6.1±5.4
Family history, hepatitis B (no.;%)	46 (59.0)	46 (56.8)	46 (59.0)
Alanine aminotransferase (IU/liter; SD)	169.6±80.0	162.5±74.7	171.8±93.4
HBV DNA (log copies/mL; SD)	7.1±0.9	7.1±0.9	7.2±0.8

SD: Standard deviation.

### Kinetics of responses in patients

As shown in Table 2, a delayed response to YIC was observed between 24 weeks and the end of follow-up. For intent-to-treat analysis, at the end of follow-up, 31, 35 and 28 patients achieved the primary endpoint, and 6, 7 and 14 patients reached the secondary endpoint in the placebo, 30 µg YIC and 60 µg YIC groups, respectively. The response rate for secondary endpoint in the 60 µg YIC group was comparatively higher than that of the other groups, though statistical significance had not been reached. However, at the end of follow-up, a significant difference on HBeAg seroconversion was found between 60 µg YIC and placebo groups (p = 0.03, 2-tailed). The 95% confidence interval for this difference was 1.5% to 24.1%. In contrast, a statistical significance had not been reached between 30 µg YIC and placebo groups.

**Table 2:** Virologic response at the end of treatment and the end of follow-up.

	End of treatment (week 24)				End of follow-up (week 44)			
	Placebo group (N = 78)	30 µg YIC group (N = 81)	60 µg YIC group (N = 78)	p value	Placebo group (N = 78)	30 µg YIC group (N = 81)	60 µg YIC group (N = 78)	p value
HBeAg loss (No.;%)	8 (10.3)	9 (11.1)	9 (11.5)	0.99	9 (11.5)	11 (13.6)	18 (23.1)	0.13
Presence of anti-HBe antibody (No.;%)	9 (11.5)	15 (18.5)	15 (19.2)	0.65	10 (12.8)	18 (22.2)	23 (29.5)	0.06
HBeAg seroconversion* (No.;%)	6 (7.7)	8 (9.9)	6 (7.7)	0.97	7 (9.0)	10 (12.3)	17 (21.8)	0.08
HBV DNA	13 (16.7)	21 (25.9)	21 (26.9)	0.46	28 (35.9)	21 (25.9)	29 (37.2)	0.28
>2log decrease (No.;%)								
Primary endpointˆ; (No.;%)	18 (23.1)	22 (27.2)	26 (33.3)	0.63	31 (39.7)	28 (34.6)	35 (44.9)	0.39
Secondary endpoint$ (No.;%)	3 (3.8)	8 (9.9)	6 (7.7)	0.59	6 (7.7)	7 (8.6)	14 (17.9)	0.14

* : 95% confidence interval (2-tailed) for the difference of response rate at week 44 between 60 µg YIC and placebo groups was 1.5% to 24.1%; it was −6.3% to 13.0% between 30 µg YIC and placebo groups.

ˆ; : 95% confidence interval (2-tailed) for the difference of response rate at week 44 between 60 µg YIC and placebo groups was −10.4% to 20.6%; it was −20.2% to 9.8% between 30 µg YIC and placebo groups.

$ : 95% confidence interval (2-tailed) for the difference of response rate at week 44 between 60 µg YIC and placebo groups was −0.2% to 20.7%; it was −7.6% to 9.5% between 30 µg YIC and placebo groups.

Furthermore, when applying the GEEs method to estimate the group and time effects, a significant difference of group effects was found between the 60 µg group and the placebo group in terms of the primary endpoint, and response rate calculated by both primary and secondary endpoints changed significantly over the time in the placebo, 30 µg and 60 µg YIC groups (p<0.05) (Table 3).

**Table 3:** Repeated measures analysis for time and group effects controlling by baseline ALT and HBV DNA.

Group	Primary endpoint		Secondary endpoint	
	OR (95% confidence interval)	p value	OR (95% confidence interval)	p value
30 µg YIC group vs placebo group	0.9 (0.5–1.4)	0.52	1.3 (0.5–3.6)	0.60
60 µg YIC group vs placebo group	1.7 (1.0–2.7)	0.04	2.2 (0.9–5.4)	0.09
Week 12 vs Week 44	0.3 (0.2–0.5)	<0.0001	0.3 (0.2–0.6)	0.0006
Week 24 vs Week 44	0.5 (0.4–0.7)	<0.0001	0.6 (0.4–0.9)	0.01
Week 36 vs Week 44	0.7 (0.6–1.0)	0.04	0.6 (0.4–1.0)	0.04

When the baseline serum HBV DNA and levels of HBeAg from all secondary responders at the end of follow-up in the three groups were analyzed, among the 14 patients immunized with 60 µg YIC , five had HBV DNA≥10^7^ copies/ml, eight had HBV DNA ≥10^6^ and <10^7^ copies/ml, only one had HBV DNA 10^5^ copies/ml. In contrast, of the 6 patients immunized with alum, five patients had HBV DNA 10^5^ copies/ml, one had HBV DNA ≥10^6^ and <10^7^ copies/ml, none of them had HBV DNA ≥10^7^ copies/ml.

### Reversion of HBeAg and rebound of HBV DNA

The incidence of rebound in virus replication and reversion to serum HBeAg at the end of follow-up were compared among the three groups. For those responders who reached the secondary endpoint, none of the patients from either the 60 µg YIC group or placebo group showed a rebound in virus load nor in reversion to serum HBeAg. In contrast, four responders in the 30 µg YIC group showed a virus load rebound to the baseline level and HBeAg reverted to positive. Interestingly, rebound of viral load and reversion to serum HBeAg were all from responders who only reached the primary endpoint at the end of immunization, while none occurred in those who reached the secondary endpoint, suggesting that patients who achieved the secondary response at the endpoint of treatment were unlikely to develop reversion.

### Adverse events and severe adverse events

Overall, the most common systematic symptoms were similar for the three groups. However, more local reactions were found in the 60 µg YIC and 30 µg YIC groups. In the placebo group, the most common reaction was pain at the injection site, followed by malaise and fatigue; in the 30 µg and 60 µg YIC group, the most common adverse events were pain at the injection site, pruritus and swelling (Table 4). During study period, eleven patients experienced elevated ALT levels over ten times the normal level, accompanied by high levels of serum bilirubin and thus were hospitalized.

**Table 4:** Occurrence of most common adverse events during treatment and follow-up.

Adverse events	No. of patients (%)			p value
	Placebo group (N = 79)	30 µg YIC group (N = 83)	60 µg YIC group (N = 80)	
Local adverse reaction				
Erythema	6 (7.6)	15 (18.1)	15 (18.8)	0.08
Swelling	9 (11.4)	19 (22.9)	25 (31.3)	0.009
Pain	32 (40.5)	42 (50.6)	45 (56.3)	0.13
Pruritus	12 (15.2)	22 (26.5)	34 (42.5)	0.0006
Systematic symptom				
Fever	11 (13.9)	8 (9.6)	11 (13.8)	0.63
Malaise	16 (20.3)	12 (14.5)	12 (15.0)	0.57
Headache	8 (10.1)	9 (10.8)	17 (21.3)	0.09
Dizziness	11 (13.9)	11 (13.3)	18 (22.5)	0.24
Fatigue	14 (17.7)	19 (22.9)	19 (23.8)	0.62
Vomiting	5 (6.3)	5 (6.0)	4 (5.0)	0.94
Nausea	8 (10.1)	9 (10.8)	14 (17.5)	0.35
Abdominal pain	6 (7.6)	6 (7.2)	8 (10.0)	0.84
Diarrhoea	6 (7.6)	6 (7.2)	4 (5.0)	0.81

The occurrence of serious adverse events calculated according to the intent-to-treat principle was 5.1% (4/79), 3.6% (3/83), and 5.0% (4/80) in the placebo, 30 µg YIC and 60 µg YIC groups respectively (p>0.05). Ten of these were males, while one was female. The age of patients with SAE varied between 21 and 41years of age. Four occurred after the first injection (2 in placebo group, 2 in 60 µg YIC group), two occurred after the second injection (1 in placebo group, 1 in 30 µg YIC group), one occurred after 5 injections (in 30 µg YIC group), and the other four appeared after 6 injections (1 in placebo group, 1 in 30 µg YIC group and 2 in 60 µg YIC group). All 11 patients were hospitalized and recovered after treatment without immunomodulating drugs (one patient used antiviral treatment). No deaths were observed during the study period.

## Discussion

In this study, statistical significant differences in the preset primary and secondary endpoints among the three groups of patients were not reached at the end of treatment or of follow up. Nevertheless, patients immunized with intramuscular injections of 60 µg YIC showed the highest rates of HBeAg loss (23.1%), HBeAg seroconversion (21.8%) and suppression of HBV DNA (37.2%) at the end of follow-up (44 weeks). When one of the primary endpoints (HBeAg) seroconversion, was compared between 60 µg YIC and placebo groups at the end of follow-up, statistical significance was observed (p = 0.03). Interestingly, these rates in 60 µg YIC group at 44 weeks markedly surpassed those achieved at 24 weeks, namely, HBeAg loss (23.1% vs 11.5%), HBeAg seroconversion (21.8% vs7.7%) and suppression of HBV DNA (37.2% vs26.9%) (Table 2). An important difference between YIC as an active immunotherapeutic vaccine versus passive immunotherapies such as using interferon, thymosin et al for treatment, is that, active immunotherapeutic approach functions through inducing immune responses in the patients; while in passive immunotherapeutic approaches, the immunological modulating products are repeatedly introduced into patients, and thus continuously providing the patients with the necessary immunological modulating products. The former approach needs only few injections at relatively long intervals, while the latter needs repeated injections of products to immunomodulate host immune responses. Therefore, it is not surprising, a late and sustained response versus YIC immunization was observed in a subpopulation of patients.

Though the response rates with respect to the secondary endpoints in the 60 µg YIC group were comparatively higher than that of the other groups, due to the unexpected rates of HBeAg seroconversion and suppression of serum HBV DNA in the alum immunized group, statistical significance was not reached. Nevertheless, after adjusting the dependence among repeated observations made on the same patient, a significant change of response rate over the study period was detected in the placebo, 30 µg and 60 µg YIC groups, with respect to either primary or secondary endpoints (p<0.05) (Table 3).

It was intriguing that only alum immunization resulted in a decrease of HBV viral load and seroconversion of HBeAg in some patients. As shown in the analysis of patients who reached secondary response at the end of follow-up, the baseline serum HBV DNA in patients who responded to alum alone immunization predominantly were those who had lower levels of serum HBV DNA (10^5^ copies/mL). Whether this phenomenon was due to spontaneous sero-conversion in patients needs to be considered. Ideally, to include a group of patients without injections as additional control in the study may well clarify this issue. However, due to ethical concern, as well as double blinding principle, such study design has not been approved. Using the data from a previous study by Yuen et al as a reference, the one year HBeAg sero-conversion rate in patients treated with IFN-α and untreated patient was 21.1% and 2.2% [25]. In another clinical trial of lamivudine treatment, at the end of one year, the HBeAg sero-conversion rate was 8.3% in the treated group [26]. These studies suggested that the 21.8% of HBeAg seroconversion rate in Chinese patients observed in this study most likely was not due to spontaneous sero-conversion. Besides, by pair-wise comparison, significance of HBeAg seroconversion rate was only found between the 60 µg YIC and the placebo groups, other than between the 30 µg YIC and the placebo groups, which further supported the previous finding was not due to spontaneous seroconversion. As for the 10 patients in the 60 µg YIC immunized group who only reached primary responses at the end of treatment, attained secondary responses during follow-up, could be due to a late response to YIC immunization, however, spontaneous HBeAg sero-conversion should be excluded. Recently, it was reported that immune complex-loaded dendritic cells were superior to soluble immune complexes as an anti-tumor vaccine in animals [27], Furthermore, antigen-antibody immune complexes were reported to empower dendritic cells to efficiently prime specific CD8+ CTL responses *in vivo* [28]. These studies strengthened our confidence in using immune complexes as a therapeutic vaccine for persistent infections. Compared to passive immunotherapy, we consider active immunization by YIC is a simple-to-use, less expensive and promising therapeutic vaccine in a subpopulation of chronic hepatitis B patients.

Regarding the effect of alum, it was reported that alum alone can promote B cell activation in mice, which could bypass the priming effect needed for B cell responses [29]. Whether alum also can induce T cell responses is still under discussion [30]. One may speculate that immunization with alum alone activated the B cells in some chronic hepatitis B patients and when these B cells came across the circulating HBsAg in these patients, a low level of immune responses to HBsAg might be triggered and eventually could lead to decrease in virus load, and /or sero-conversion of HBeAg.

Patients who developed severe adverse events were distributed almost equally in the three groups. It was surprising that three patients immunized with only alum developed severe adverse events, and two of them even developed severe adverse events after only one injection. The severe adverse events in these two patients were verified by highly elevated ALT levels attaining 937 U/L and 818 U/L with TB 111.6 µmol/L and 54.8 µmol/L respectively. In the forthcoming phase III clinical trial, not only the therapeutic efficacy of YIC should be evaluated, but also severe adverse events will be closely monitored and investigated.

## Supporting Information

Protocol S1 Trial Protocol.
(0.49 MB DOC)

Checklist S1 CONSORT Checklist.
(0.06 MB DOC)
