# Correction: A Randomized Controlled Phase IIb Trial of Antigen-Antibody Immunogenic Complex Therapeutic Vaccine in Chronic Hepatitis B Patients

**DOI:** 10.1371/annotation/5abf9f11-280c-4682-9b95-001435014303

**Published:** 2008-08-04

**Authors:** Dao-Zhen Xu, Kai Zhao, Li-Min Guo, Lan-Juan Li, Qin Xie, Hong Ren, Ji-Ming Zhang, Min Xu, Hui-Fen Wang, Wen-Xiang Huang, Xue-Fan Bai, Jun-Qi Niu, Pei Liu, Xin-Yue Chen, Xin-Liang Shen, Zheng-Hong Yuan, Xuan-Yi Wang, Yu-Mei Wen

The Authors are listed out of order. Please view the correct author order, affiliations, and citation here:

Dao-Zhen Xu^1^, Kai Zhao^2^, Li-Min Guo^1^, Lan-Juan Li^3^, Qin Xie^4^, Hong Ren^5^, Ji-Ming Zhang^6^, Min Xu^7^, Hui-Fen Wang^8^, Wen-Xiang Huang^9^, Xue-Fan Bai^10^, Jun-Qi Niu^11^, Pei Liu^12^, Xin-Yue Chen^13^, Xin-Liang Shen^2^, Zheng-Hong Yuan^14^, Xuan-Yi Wang^14,15^, Yu- Mei Wen^14^

1 Ditan Hospital, Beijing, China, 2 Beijing Institute of Vaccine and Biological Products, Beijing, China, 3 First affiliated hospital, Zhejiang University, Hangzhou, Zhejiang Province, China, 4 Rui-Jin Hospital, Shanghai Jiaotong University, Shanghai, China, 5 Second affiliated hospital, Chongqing Medical University, Chongqing, China, 6 Hua-Shan Hospital, Fudan University, Shanghai, China, 7 Guangzhou Eighth Hospital, Guangzhou, Guangdong Province, China, 8 302 Military Hospital, Beijing, China, 9 First affiliated hospital, Chongqing Medical University, Chongqing, China, 10 Tangdu Hospital, Fourth Military Medical University, Xi’an, Shanxi Province, China, 11 First affiliated hospital, Jilin University, Changchun, Jilin Province, China, 12 Second hospital affiliated to China Medical University, Shenyang, Liaoning Province, China, 13 You-An Hospital, Capital Medical University, Beijing, China, 14 Key Laboratory Medical Molecular Virology, Shanghai Medical College, Fudan University, Shanghai, China, 15 Institute of Biological Sciences, Fudan University, Shanghai, China

Xu D-Z, Zhao K, Guo L-M, Li L-J, Xie Q, et al. (2008) A Randomized Controlled Phase IIb Trial of Antigen-Antibody Immunogenic Complex Therapeutic Vaccine in Chronic Hepatitis B Patients. PLoS ONE 3(7): e2565. doi:10.1371/journal.pone.0002565

