# Correction: A Randomized Controlled Phase IIb Trial of Antigen-Antibody Immunogenic Complex Therapeutic Vaccine in Chronic Hepatitis B Patients

**DOI:** 10.1371/annotation/8b913538-74f4-4560-b700-0936a8e35847

**Published:** 2008-08-13

**Authors:** Dao-Zhen Xu, Kai Zhao, Li-Min Guo, Lan-Juan Li, Qin Xie, Hong Ren, Ji-Ming Zhang, Min Xu, Hui-Fen Wang, Wen-Xiang Huang, Xue-Fan Bai, Jun-Qi Niu, Pei Liu, Xin-Yue Chen, Xin-Liang Shen, Zheng-Hong Yuan, Xuan-Yi Wang, Yu-Mei Wen

The author name Wen-Xiang Wang is spelled incorrectly. The correct spelling is: Wen-Xiang Huang.

