# Supplementary material for: A Randomized Controlled Phase IIb Trial of Antigen-Antibody Immunogenic Complex Therapeutic Vaccine in Chronic Hepatitis B Patients
Source: PLoS One. 2008 Jul 2;3(7):e2565. doi: 10.1371/journal.pone.0002565 (PMC2430617; doi:10.1371/journal.pone.0002565)
Supplement: Protocol S1 — Trial Protocol. (0.49 MB DOC) [file pone.0002565.s001.doc]

# **
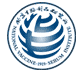
** **
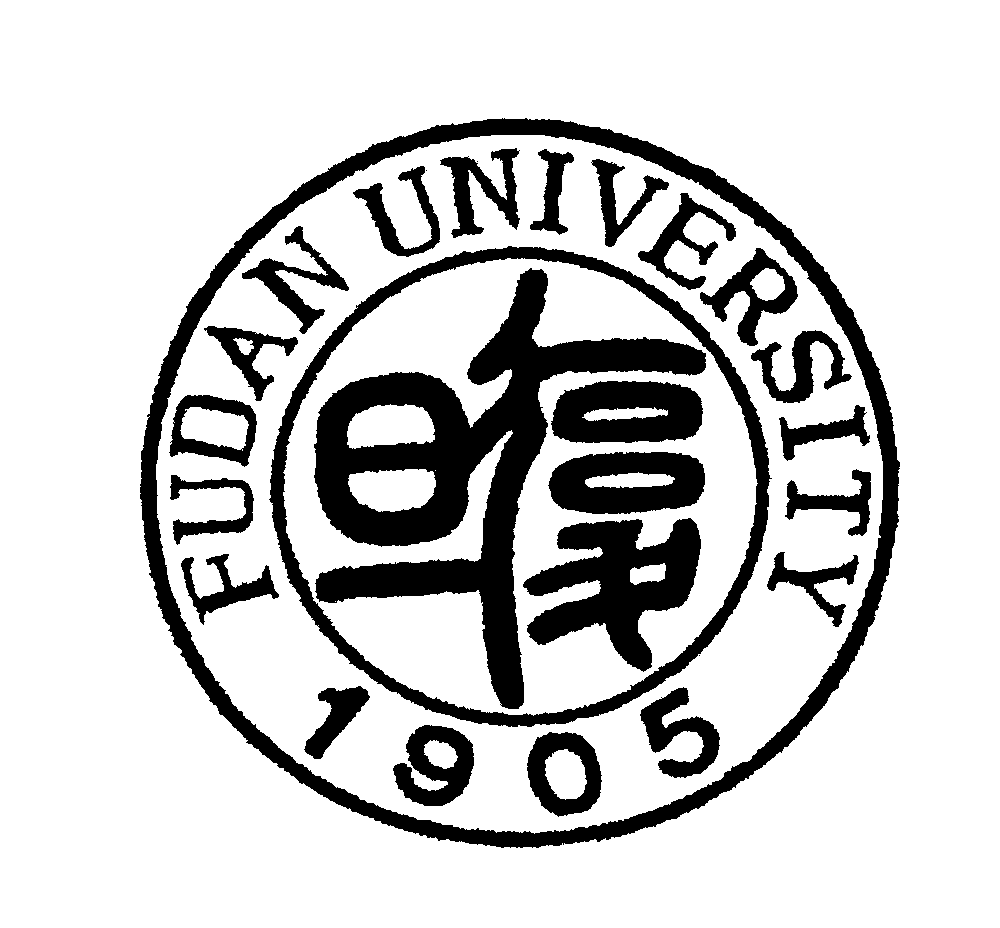

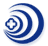

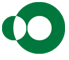
**

**Trial Working Protocol**

**Project Title**

**Efficacy, safety of therapeutic hepatitis B vaccine (YIC) among chronic hepatitis B patients: a multi-center, randomized, double-blinded, controlled phase IIB trial**

| **File name: TY0508YIC**  **Version: 2.0**  **Finalized: October 24, 2005** |
| --- |

# **[[1]](#endnote-2)**

***= CONFIDENTIAL =***

| **Protocol No.：** | TG0508YIC |
| --- | --- |
| **Date：** | October 24, 2005 |
| **Version：** | 2^nd^ |
| **SFDA Registry No.：** | 2002SL0038 |
| **Trial Registry No.:** | ChiCTR-TRC-00000022 |
| **SPONSOR：** | Beijing Vaccine Institute, and Shanghai Medical College, Fudan University |
| **Principle Investigator：** | Ditan Hospital, Beijing |
| **CRO：** | Shanghai Tigermed Consulting Ltd. |
| **Statistic analyst：** | Department of Biostatistics, Shanghai Second Medical University |

**STUDY SYNOPSIS**

| Protocol No. | TG0508YIC |
| --- | --- |
| Title | Efficacy, safety of therapeutic hepatitis B vaccine (YIC) among chronic hepatitis B patients: a multi-center, randomized, double-blinded, controlled phase IIB clinical trial |
| Version | 2^nd^ version, Oct 24, 2005 |
| SPONSOR | Beijing Vaccine Institute, and Shanghai Medical College, Fudan University |
| Phase | Phase IIB |
| Study population | Chronic hepatitis B patients |
| Objective | - To study the tolerance and efficacy of YIC among chronic hepatitis B patients through the comparison between 30 μg, 60 μg YIC and placebo groups. - To study the optimal regimen |
| Study design | Double-blinded, randomized, placebo-controlled trial with basic treatment. Subjects will be assigned to 30 μg, 60 μg YIC and placebo group equally. |
| Sample size | 240 patients |
| Number of study center | 12 |
| Duration | 44 weeks (0- 20 weeks treatment plus 24 weeks follow-up) |
| Selection Criteria | Inclusion criteria:   1. Patients with chronic hepatitis B aged 18 to 65 years; 2. Patients with > 6 months HBsAg positive prior to study; 3. HBeAg positive, HBeAb negative, and HBV DNA > 1×10^5^ copies/ml; 4. Patients with ALT abnormal (2-fold higher but 10-fold less than cutoff at baseline.)   Exclusion criteria:   1. Co-infection with hepatitis A, C, D and E virus, or HIV; 2. Taking antiviral, hepatotoxic or immunosuppressive drugs or products within the preceding 6 months; 3. Other causes of liver disease; 4. Serious medical or psychiatric illness; 5. Hepatic cirrhosis or AFP>100 ng/ml; 6. Abnormal serum creatinine, thrombocyte count, 7. Hematoglobin or serum total bilirubin abnormal; 8. And pregnancy |
| Study agent | Vaccine:：therapeutic hepatitis B vaccine, 60 μg per ampoule.  Placebo：0.1% alum, 1 mL per ampoule |
| Study arm | Three arms, 80 cases for each.  Vaccine groups: 30 μg YIC and 60 μg YIC  Control group: placebo |
| Endpoints | The virologic response was assessed four weeks after the end of treatment (week 24) and at the end of follow-up (week 44). HBeAg seroconversion was defined by the loss of HBeAg and the presence of anti-HBe antibody. Suppression of HBV DNA was defined as > 2 log10 decrease of viral load.   - The primary endpoint was defined as loss of HBeAg, or presence of anti-HBe antibody or suppression of HBV DNA. - The secondary endpoint was designated as both HBeAg seroconversion and suppression of HBV DNA. |
| Safety endpoints | Adverse events, laboratory testing, systemic and local reactions. |
| Study procedure | - After the protocol being approved by the ethic committee, and inform consenting, patients will be screened and assigned to 30 μg, 60 μg YIC and placebo groups randomly; - Study agents will be given at baseline (0 week), 4^th^, 8^th^, 12^th^, 16^th^ and 20^th^ week; - Treatment will be ended at 20^th^ week, and followed both at 24^th^ week and by 44^th^ -week for efficacy follow-up; - Subjects will be interviewed at baseline, 4^th^, 8^th^, 12^th^, 16^th^, 20^th^, 24^th^, 36^th^, and 44^th^ week. Each interview will include blood cell count and biochemical liver function testing. Virologic and HBeAg/anti-HBe from samples of all patients will be assayed at the same time for serum at baseline, 12^th^, 24^th^, 36^th^, and 44^th^ week at the reference lab after 44-week follow-up. - Adverse events, and concomitant medication will be recorded at each interview. |

**TABLE OF CONTENTS**

[1 STUDY TITLE 9](#__RefHeading___Toc187041360)

[2 Rationale 9](#__RefHeading___Toc187041361)

[**2.1 General Introduction 9**](#__RefHeading___Toc187041362)

[**2.2 Rationale of dosage 10**](#__RefHeading___Toc187041363)

[3 Objectives 11](#__RefHeading___Toc187041364)

[4 Study Disgn 11](#__RefHeading___Toc187041365)

[**4.1 Study agent 11**](#__RefHeading___Toc187041366)

[**4.1.1 Vaccine 11**](#__RefHeading___Toc187041367)

[**4.1.2 Placebo 11**](#__RefHeading___Toc187041368)

[**4.2 Study population 11**](#__RefHeading___Toc187041369)

[**4.2.1 Inclusion criteria 11**](#__RefHeading___Toc187041370)

[**4.2.2 Exclusion criteria 12**](#__RefHeading___Toc187041371)

[**4.3 Study procedure 13**](#__RefHeading___Toc187041372)

[**4.3.1 Informed consent 13**](#__RefHeading___Toc187041373)

[**4.3.2 Screening (two weeks prior to treatment) 13**](#__RefHeading___Toc187041374)

[**4.3.3 Baseline visit (week 0), and vaccination 14**](#__RefHeading___Toc187041375)

[**4.3.4 Visits during the treatment period (Week 4, 8, 12, 16, and 20 ± 4 days after the initial dose) 15**](#__RefHeading___Toc187041376)

[**4.3.5 Visits at the end of treatment (Week 24 ± 4 days） 16**](#__RefHeading___Toc187041377)

[**4.3.6 Visit at the end of basic treatment and follow-up（Week 36/44 ± 4 days） 16**](#__RefHeading___Toc187041378)

[**4.3.7 Blinding and Unblinding 17**](#__RefHeading___Toc187041379)

[**4.3.8 Discontinuation/Withdrawal 18**](#__RefHeading___Toc187041380)

[**4.4 Laboratory assay 18**](#__RefHeading___Toc187041381)

[**4.4.1 Reference lab 18**](#__RefHeading___Toc187041382)

[**4.4.2 Clinical laboratory at study centers 19**](#__RefHeading___Toc187041383)

[**4.4.3 Testing during follow-up period 19**](#__RefHeading___Toc187041384)

[**4.5 Safety measurement 19**](#__RefHeading___Toc187041385)

[**4.5.1 Definition of adverse events 20**](#__RefHeading___Toc187041386)

[**4.5.2 Surveillance of adverse events following injections 20**](#__RefHeading___Toc187041387)

[**4.5.3 Recording adverse events 21**](#__RefHeading___Toc187041388)

[**4.5.4 Criteria of adverse events intensity 21**](#__RefHeading___Toc187041389)

[**4.5.5 Criteria of adverse events causality 21**](#__RefHeading___Toc187041390)

[**4.5.6 Measures taken after abnormal laboratory tests 22**](#__RefHeading___Toc187041391)

[**4.5.7 Treatment and follow-up of adverse events 22**](#__RefHeading___Toc187041392)

[**4.5.8 Definition for serious adverse event (SAE) 23**](#__RefHeading___Toc187041393)

[**4.5.9 Unscheduled pregnancy 23**](#__RefHeading___Toc187041394)

[**4.5.10 Recording and reporting of SAE 23**](#__RefHeading___Toc187041395)

[**4.5.11 Important noticeable event 24**](#__RefHeading___Toc187041396)

[**4.5.12 Procedure for causal relationship assessment 24**](#__RefHeading___Toc187041397)

[**4.6 Efficacy measurement 24**](#__RefHeading___Toc187041398)

[**4.6.1 Definition of endpoints 24**](#__RefHeading___Toc187041399)

[**4.6.2 Primary endpoint 25**](#__RefHeading___Toc187041400)

[**4.6.3 Secondary endpoint 25**](#__RefHeading___Toc187041401)

[**4.7 Basic treatment 25**](#__RefHeading___Toc187041402)

[**4.8 Concomitant medication 25**](#__RefHeading___Toc187041403)

[5 Data management 26](#__RefHeading___Toc187041404)

[**5.1 The System 26**](#__RefHeading___Toc187041405)

[**5.2 Data checking and coding 26**](#__RefHeading___Toc187041406)

[**5.3 Data checking, data entry and error checking 26**](#__RefHeading___Toc187041407)

[**5.4 Data freezing 27**](#__RefHeading___Toc187041408)

[6 Statistical Methods 27](#__RefHeading___Toc187041409)

[**6.1 Safety 27**](#__RefHeading___Toc187041410)

[**6.2 Efficacy 27**](#__RefHeading___Toc187041411)

[**6.3 Sample size 28**](#__RefHeading___Toc187041412)

[7 Coding and randomization 28](#__RefHeading___Toc187041413)

[8 Packaging and labeling 29](#__RefHeading___Toc187041414)

[**8.1 Packaging 29**](#__RefHeading___Toc187041415)

[**8.2 Labeling 29**](#__RefHeading___Toc187041416)

[9 Handling and distribution of study agent 30](#__RefHeading___Toc187041417)

[10 ETHICAL CONSIDERATIONS 30](#__RefHeading___Toc187041418)

[**10.1 Protocol Review 30**](#__RefHeading___Toc187041419)

[**10.2 Ethical Guidelines 31**](#__RefHeading___Toc187041420)

[**10.3 Informed Consent 31**](#__RefHeading___Toc187041421)

[**10.4 Confidentiality 31**](#__RefHeading___Toc187041422)

[**10.5 Potential Risks and Risk Minimization 31**](#__RefHeading___Toc187041423)

[**10.5.1 Vaccination 31**](#__RefHeading___Toc187041424)

[**10.5.2 Blood collection 32**](#__RefHeading___Toc187041425)

[**10.6 Protocol Modification 32**](#__RefHeading___Toc187041426)

[**10.7 Stipends for Participation 32**](#__RefHeading___Toc187041427)

[**10.8 Compensation 32**](#__RefHeading___Toc187041428)

[11 Compliance, monitoring, and archiving 32](#__RefHeading___Toc187041429)

[12 PUBLICATION POLICY 34](#__RefHeading___Toc187041430)

[13 References 36](#__RefHeading___Toc187041431)

[Appendix 1. List of abbreviations 37](#__RefHeading___Toc187041432)

[appendix 2. Flow chart 39](#__RefHeading___Toc187041433)

[Appendix 3. WORLD MEDICAL ASSOCIATION DECLARATION OF HELSINKI 41](#__RefHeading___Toc187041434)

[Appendix 4. THE PRINCIPLES OF ICH GCP 47](#__RefHeading___Toc187041435)

# STUDY TITLE

Efficacy, safety of therapeutic hepatitis B vaccine (YIC) among chronic hepatitis B patients: a multi-center, randomized, double-blinded, controlled phase IIB clinical trial

# Rationale

- 1. General Introduction

Viral hepatitis B is highly endemic in China, with estimated 130 million chronic hepatitis B surface antigen carriers. Of these, 30 million infected individuals are chronic active hepatitis patients who need antiviral therapy, which mostly relies on interferon α and lamivudine. The short-term efficacy response to interferon α is 40-60%, which reduces to 20-40% one year after the treatment, and its use is associated with dose-related side effects. For lamivudine, after one year consecutive treatment, the loss of HBeAg reaches 20-25%, and HBeAg seroconversion rate is 15-20%. However, the use of lamivudine for more than 6 months is associated with the emergence of resistant YMDD variants. Furthermore, rebounds of HBV DNA are frequently observed after longer period of treatment. Hence, effective immuno-modulating drugs or products to enhance host immune responses aiming to break immune tolerance to hepatitis B virus (HBV) is encouraged.

The therapeutic hepatitis B vaccine (Yeast derived Immunogenic Complex, YIC), consisting of immune complexes of HBsAg and high titer of human anti-HBs immunoglobulin (HBIG) at an appropriate ratio, is developed by the Key Laboratory Medical Molecular Virology, Shanghai Medical College, Fudan University with the financial support of 863 National Program. It possesses Chinese patent**.** Pre-clinical animal experiments revealed that, YIC was able to clear circulating HBeAg, induce anti-HBs, INF-γ and CTL in transgenic mice. The YIC vaccine was formulated under GMP by Beijing Vaccine Institute and has passed the qualification by the National Institute for the Control of Pharmaceutical and Biological Products. The GCP-compliant phase I and phase IIa clinical trials was conducted by Beijing Ditan Hospital in 2003 and 2004 respectively. The safety and tolerance of 30 μg、60 μg, and 90 μg YIC with 3-dose regimen and 6-dose regimen of 90 μg YIC have been proved in phase I trial. Subsequently, 36 chronic hepatitis B patients were assigned to placebo group, 60 μg and 90 μg YIC groups randomly in Phase IIa trial. All participants were immunized with six intramuscular injections at 4- week intervals, and followed to 44^th^ week. A basic treatment (Silymarin, 50 mg per tablet, 3 tablets per time, tid; and Hu Gan Pian tablet, an extraction of Chinese herb containing Wu Wei Zi) which has proved to be effective for improving liver function, 0.36 g per tablet, 4 tablets per time, tid) was given to each participant of all groups throughout the 44-week period. After the termination of immunization, serum samples were collected from each patient at baseline, 12th, 24th, 36th and 44th week after initial injection for assay of HBeAg, anti-HBe, HBV DNA, and alanine aminotransferase (ALT). Because immune responses to immunotherapy should be monitored some time later after the last injection, four weeks after the end of treatment (24^th^ week)and follow-up for 24 weeks ( 44 week) were set for the time points in this study. Suppression of HBV DNA (less than 1.0*10^5^ copies/mL) was found in 4 patients, one was in the placebo group, the other three were in 60 μg YIC group, none was found in 90 μg YIC group. HBeAg seroconversion was found in two patients in 60 μg YIC group. The most common adverse event in phase IIa trial was local reaction at injection site, .Almost all adverse events were mild. In some participants temporary elevated ALT was observed, which recovered to normal levels at follow up. Serious adverse event was not observed.

- 1. Rationale of dosage

In terms of the conclusion of phase IIA, efficacy of 60 μg YIC group was better than 90 μg YIC and placebo groups. However, phase IIA trial was not able to exclude the possibility that 30 μg YIC might be the optimal dosage. Therefore, both 30 μg and 60 μg YIC will be included in the phase IIB study to work out the optimal dosage through the comparison with placebo control.

# Objectives

- To study the tolerance and efficacy of YIC among chronic hepatitis B patients through the comparison between 30 μg, 60 μg YIC and placebo groups.
- To optimize dosage and regimen

1. Study Disgn

This is a double-blinded, randomized, placebo-controlled trial with basic treatment.

- 1. Study agent

### Vaccine

Therapeutic hepatitis B vaccine (commercial name: YIC, Lot: 20050501), 30 μg or 60 μg per mL per ampoule, manufactured by Beijing Vaccine Institute, and stored at 2 to 8^o^C.

### Placebo

Placebo consists of 0.1% alum adjuvant, 1 mL per ampoule. It is manufactured by Beijing Vaccine Institute with the identical appearance of vaccine.

- 1. Study population

Chronic hepatitis B patients will be evaluated and recruited at each treatment center in the trial. Following inclusion and exclusion criteria will be employed for eligibility evaluation.

### Inclusion criteria

- 1. Patients provide written inform consent;
  2. Patients aged 18 to 65years regardless of gender;
  3. Patients with HBsAg positive in the preceding 6 months;
  4. Patients with HBeAg positive, HBeAb negative, and virus load (HBV DNA) > 1×10^5^ copies/ml;
  5. Patients with abnormal ALT (between 2- and 10-fold of cutoff value). If abnormal ALT had appeared in the preceding 6 months, the interval period should be longer than one month;
  6. For reproductive age women, the uric or blood pregnancy test should be negative. Both male and female patients should not have birth plan until 3 months after the completion of study.

### Exclusion criteria

1. Pregnancy women or lactation women;
2. Patients who have received antiviral therapy or immunosuppressive drugs, such as adrenal cortical hormone, thymic peptide α1 in the preceding 6 months or long-term;
3. Patients co-infected with HAV, HCV, HDV, HEV and HIV;
4. Patients with history of or current allergic diseases, hypersensitivity to any biological product injection, and autoimmune diseases;
5. Patients who have received hepatotoxic drugs in the preceding 6 months;
6. Patients with confirmed or suspected liver cirrhosis, or compensated cirrhosis;
7. Patients with confirmed or suspected primary hepatic carcinoma or AFP>100ng/ml;
8. Patients with other liver diseases, including chronic alcoholic hepatitis, drug-induced hepatitis, autoimmune hepatitis（antinuclear antibody > l:100）、Kinnier-Wilson syndrome, and hemachromatosis;
9. Life-threatening or serious cardiac, respiratory, gastrointestinal, renal, endocrine, hematological or immunological disorders which, in the opinion of the investigator, will be **excluided** in the study;
10. Serum creatinine > 1.5-fold of upper limit of cutoff value;
11. Blood platelets count < 75×10^9^/L；
12. Leucocyte count < 3×10^9^/L；
13. Haematoglobin <11.5g/dL（female） or <12.5g/dL（male）;
14. Serum total bilirubin > 2-fold of upper limit of normal value;
15. Patients with history of drug addiction, including alcohol abuse (40g/day) in the preceding 6 months;
16. Patients had participated in other clinical trials in the preceding 3 months.
    1. Study procedure

The core procedures see appendix 2. The entire study duration will be 46 weeks, including 2-week run-in, 20-week treatment and 24-week follow-up.

### Informed consent

Informed consent must be obtained from each potential patient to be enrolled in the study. The information in the consent form should be communicated to the patient in language that they can understand. The consent form and any subsequent revisions must be reviewed by the Institutional Review Board (IRB) overseeing the study.

### Screening (two weeks prior to treatment)

After signing the informed consent,, every potential participant will be assigned a screening number. Screening will be performed in a two-week period prior to the treatment in terms of the inclusion/exclusion criteria. Following investigation of medical history, including family history on hepatitis B infections, and physical examination (temperature, heart rate, and blood pressure), a series of tests will be performed:

1. X-ray[[2]](#footnote-2), type B ultrasonic (liver, spleen and gallbladder)[[3]](#footnote-3), electrocardiogram;
2. Urine or blood HCG pregnancy test[[4]](#footnote-4);
3. Serum HBeAg、HBsAg、HBsAb 、HBeAb、HBcAb;
4. Liver function, including ALT、AST、TBil、DBil、TP、ALB、AKP;
5. Biochemistry tests, including blood cell count, microscopy[[5]](#footnote-5), uric tests^d^, renal function and electrolytes;
6. PTA;
7. Blood glocuse;
8. Anti-HAV IgM, anti-HDV，and anti-HEV;
9. Anti-HCV, and anti-HIV;
10. Antinuclear antibody;
11. AFP
12. HBV DNA (PCR fluorescent quantitation)

Ten mL blood sample from each screened patient will be required. The serum will be distributed into two vials (2 mL for each), and kept at minus 70 centigrade freezer for repeating assay at reference lab.

### Baseline visit (week 0), and vaccination

#### Baseline visit

After the patient’s medical history and informed consent have been obtained, the baseline physical examination (temperature, heart rate, and blood pressure) will be performed prior to randomization and vaccination. Patients who meet the eligibility requirements for enrollment will be assigned allocation number to patients in terms of the sequence of recruitment.

#### Vaccination

Study agents will be administrated to each patient according to the allocation number on the label of study agent.

Precaution

1. Products should be shaken thoroughly prior to administration;
2. Products should be inspected visually for particulate matter or discoloration prior to administration;
3. Epinephrine should be available for potential immediate allergic reactions;
4. Injection should be carried out by experienced and per-designated personnel;
5. All used study agent-related things including empty ampoules, packages and labels should be kept;
6. To ensure the accuracy of dosage, one mL syringe should be used for injection.

Study agent code will be assigned strictly to patient in terms of the recruitment sequence. All instruction on the label of study agent will be followed.

Administration

With thorough agitation, 1 mL (for either 30 μg YIC group or 60 μg YIC group) vaccine will be drawn from ampoule using 1 mL syringe, and will be injected to patient in the deltoid region. The injection will be given at day 1, week 4, 8, 12, 16 and 20.

With thorough agitation, 1 mL placebo will be drawn from ampoule using 1 mL syringe, and will be injected to patient in the deltoid region. The injection will be given at day 1, week 4, 8, 12, 16 and 20.

A total of 240 chronic hepatitis B patients will be recruited, and assigned to 30 μg (group 2), 60 μg YIC (group 3), and placebo group (group 1) in a ratio of 1:1:1.

The immediate reaction within 30 minutes after injection will be observed. Concurrently, basic medication (Silymarin and Hu Gan Tablet) will be distributed to each participated patient. To record the solicited and non-solicited reaction in the following 4 weeks after injection, diary card will be distributed. Corresponding instruction for filling in out will be given by investigator.

### Visits during the treatment period (Week 4, 8, 12, 16, and 20 ± 4 days after the initial dose)

During the treatment period, interview and physical examination (temperature, heart rate, and blood pressure) will be conducted at week 4, 8, 12, 16, 20 and 24. Concurrently, a serials test will be performed for each recruited patients, including liver function (ALT、AST、TBil、DBil、TP、ALB、and AKP), biochemical tests, blood cell count, microscopy^d^, uric tests^d^, renal function and assays for electrolytes.

Two vials of serum sample (2 mL for each) will only be required from each patient at week 12. The serum samples will be stored at minus 70 centigrade freezer for virologic response testing at reference lab at the end of study.

The same study agent will be administrated to each patient once every four weeks till week 20. The immediate reaction within 30 minutes after injection will be observed at research centers. A diary card to record the solicited and non-solicited reaction in the following 4 weeks after each injection will be distributed to patient with the basic medication. The diary card from the pervious vaccination will be collected at the beginning of each injection.

### Visits at the end of treatment (Week 24 ± 4 days）

At the end of treatment, except for the physical examination (temperature, heart rate, and blood pressure) and all tests described above, AFP will be tested when it is necessary.

The diary card for the injection at week 20 will be collected from each patient. Ten mL blood sample will be collected and separated into 2 vials (2 mL per vial). Serum sample will be kept at minus 70 centigrade freezer for virologic response testing at reference lab in the end of study.

### Visit at the end of basic treatment and follow-up（Week 36/44 ± 4 days）

Twice visits will be conducted at week 36 and 44 during the follow-up period. Following procedures will take place during each interview:

1. Physical examination (temperature, heart rate, and blood pressure）;
2. Liver function, including ALT、AST、TBil、DBil、TP、ALB、AKP;
3. Biochemistry tests, including blood cell count, microscopy^d^, uric tests^d^, renal function and electrolytes;
4. AFP when it is needed;
5. Collect 2 vials serum sample (2 mL for each) and keep at minus 70 centigrade freezer for virologic response testing at reference lab at the end of study;
6. Collect and distribute diary card to record serious adverse event between two visits.

### Blinding and Unblinding

#### Blinding

This is a double blind (with in-house blinding) clinical trial. Study blinding is necessary to ensure the integrity of the data being collected. However, study blinding must not compromise the safety of the patients participating in the trial. The patient, investigator, and clinical personnel monitoring the study data are blinded to the study agent (30 μg YIC, 60 μg YIC and placebo). All personnel including the SPONSOR and the internal and external personnel working with the SPONSOR will remain blinded to the study agent assignment of each patient until the completion of the study.

#### Emergency unblinding

A sealed envelope which contains the individual randomization information for each randomized patient prepared by the independent biostatistician will be distributed with study agents to corresponding study centers. The envelope must be kept by pre-designated co-investigator at each center. Except in the case of medical necessity, such as serious adverse events, a patient’s treatment should not be unblended without the approval of co-principle investigator. The sealed envelope should be used ONLY if absolutely essential for the welfare of the patient.

If any patient is unblinded prior to completion of the study, the investigator must promptly contact the CRO, and document the circumstances on the envelope and sign the name of the person who unblinds the treatment. Once the unblinding occurs, the unblinded patient will be classified to lost-to-follow-up. The disclosure or masked envelopes held by the co-investigator at each center will be collected at the end of the study to ensure that the unblinding was properly documented. The sealed envelopes must be checked by clinical monitor at each visit.

#### Endpoint unblinding

All patients who completed the full treatment will be unblinded at the end of follow-up (Week 44). Two-step unblinding method will be applied. After data freezing, the patients will be classified to group A, B and C for safety and efficacy endpoint analysis. When safety and efficacy of each group are derived, the assignment of group A, B and C will be given.

### Discontinuation/Withdrawal

Any patient who had signed a consent form, and meet the following criteria will be defined as premature withdrawal.

1. Patients who request to quit themselves;
2. Patients who violate the study protocol;
3. Patients who become pregnancy;
4. Patients who are unblinded due to SAE;
5. Patients during the treatment period who developed the following symptoms
   - - - Hepatic cirrhosis
       - Hepatic failure
       - And hepatoma.

If the withdrawal occurs due to the adverse events or abnormal laboratory tests, details should be recorded in the CRFs.

- 1. Laboratory assay

### Reference lab

In order to ensure a standardized and qualified clinical assay, the clinical laboratory of Beijing Ditan Hospital will be assigned as the reference lab for this study. Serum samples collected during the study period should be transported to reference lab for repeating / testing by airway at the end of study.

Hepatitis B virus markers (HBsAg, HBeAg and HBeAb) and virus load (HBV DNA) will be tested at the reference lab. Hepatitis B virus makers will be assayed by Abbott reagent (Abbott Axisim), and the virus load will be determined by real-time PCR **(**PiJi, Shenzhen Co, China with a detection limit of 500 copies/mL**).**

### Clinical laboratory at study centers

Each patient will be screened in the corresponding study center before they are enrolled. The screening testing will be done at clinical laboratory at each center, and will be repeated at the end of the study at the reference lab. The results of screening will be employed as baseline information. The following tests will be carried out during screening at clinical lab of each center:

1. HBeAg、HBsAg、HBsAb 、HBeAb、HBcAb
2. HBV DNA quantity (real-time PCR)
3. Anti-HCV, anti-HIV, anti-HBc
4. AFP
5. Liver function: ALT, AST, TBil, DBil, TP, ALB, and AKP
6. PTA
7. Renal function: BUN, Cr, and Electrolytes (K、Na、Cl)
8. Blood cell count: WBC, RBC, Hb, and PLT
9. Uric testing

### Testing during follow-up period

In each study center, liver function, renal function, Electrolytes, blood cell count and uric testing will be performed for all enrolled patients at week 4, 8, 12, 16, 20, 24, 36 and 44.

## Safety measurement

Systemic symptoms, local reactions and allergic reactions will be detected within 30 minutes after each injection at each research center, and through diary card between two visits for all enrolled patients.

1. Local reactions: swelling, pain, edema and redness at inject site;
2. Systemic symptoms: allergic reactions, fever, erythra, pruritus, nettle rash, joint pain, **a**rthritis, obvious abnormal of hemogram, gastrointestinal reactions, renal function abnormal, autoimmune disorder, hypersensitive response.

### Definition of adverse events

*Adverse event following immunization (AEFI)*

A medical incident that takes place after vaccination, causes concern and is believed to be caused by the immunization. Reported adverse events may be either causally or coincidentally related to the vaccination.

*Coincidental AEFI*

A medical incident that would have occurred whether the individual had received an immunization prior to the incident or not.

### Surveillance of adverse events following injections

A surveillance system will be implemented to document the safety profile of the study agents during the study period. Surveillance of vaccine safety will be carried out following the WHO guidelines. At each study center, all adverse events either observed by the investigator or reported by the patients spontaneously will be evaluated by the investigator. Type III allergic reactions, such as fever, redness, pruritus, rash, joint pain and arthritis should be included in the AEs observation in this study.

The following surveillance methods will be applied to detect and record adverse events:

*Observation 30 minutes after vaccination*

All patients will be asked to remain at the vaccination site for 30 minutes immediately after vaccination to observe the immediate adverse events. Immediate serious adverse events will be treated following the WHO guidelines and recorded in the Serious Adverse Event Report Form by trained investigators. Each study center will be equipped with basic emergency equipment in case of an immediate SAE.

*Adverse events between two injections*

The potential AEs occurs between two visits will be recorded to the diary card by patients. At the beginning of each visit, investigators should query patients about AEs may occur during the two visits, and collect the diary card from last visit.

### Recording adverse events

All adverse events will be recorded as an adverse event on the adverse event forms, irrespective of severity of whether or not they are considered vaccination-related. The nature of each event, date and time (where appropriate) of onset, outcome and intensity should be established using clear medical terms.

### Criteria of adverse events intensity

When reporting adverse events, the intensity will be described as mild, moderate and severe according to the following criteria:

1. Mild: easily tolerated; causing minimal discomfort; not interfering normal everyday activities;
2. Moderate: Sufficiently discomforting to interfere with normal everyday activities;
3. Severe: Prevents normal everyday activities

Please notice the difference between the term “severe” and term “serious”. They are not synonymous. For regulatory definition, “serious” is used to describe the “seriousness”, and “severe” relates to “intensity”. Headache could be severe, but could not be SAE, unless it meets the SAE criteria.

### Criteria of adverse events causality

Causality of adverse events to injections and concomitant medication should be established by investigators according to following criteria:

1. Very likely/certain

A clinical event with a plausible relationship to vaccine administration and which cannot be expected by concurrent disease or other drugs or chemicals.

1. Probable

A clinical event with a reasonable time relationship to vaccine administration; is unlikely to be attributed to concurrent disease or other drugs or chemicals.

1. Possible

A clinical event with a reasonable time relationship to vaccine administration, but which could also be explained by concurrent disease or other drugs or chemicals.

1. Unlikely

A clinical event whose time relationship to vaccine administration makes a causal connection improbable, but which could be plausibly explained by underlying disease or other drugs or chemicals.

1. Unrelated

A clinical event with an incompatible time relationship and which could be explained by underlying disease or other drugs or chemicals.

1. Unclassifiable

A clinical event with insufficient information to permit assessment and identification of the cause.

### Measures taken after abnormal laboratory tests

When unexplainable abnormal laboratory test appears, the test must be repeated immediately , the patients should be followed-up constantly till either the abnormal test has resolved, or could be explained from investigator’s point of view. The definitive explanation should be recorded in the CRFs.

### Treatment and follow-up of adverse events

Any adverse event must be treated with acceptable method. Investigators must report and discuss with SPONSOR when a method repelling with study agent has to be implemented, since this activity will cause premature withdrawal. All adverse events should be followed-up until the event has: resolved or subsided or stabilized or disappeared or the event is otherwise explained.

### Definition for serious adverse event (SAE)

Serious adverse event is defined as any untoward medical occurrence that:

1. Results in death
2. Requires initial or prolonged inpatient hospitalization
3. Is life-threatening
4. Results in severe or permanent disability

### Unscheduled pregnancy

Any pregnancies which occur during study period should be reported to corresponding investigator, and all study related-treatment should be stopped consequently. Investigator should report to both CRO and SPONSOR using specific-pregnancy form. The pregnant patient should be followed-up till delivery, the outcome of pregnancy and delivery should be reported to both CRO and SPONSOR. The pregnancy occurs with participant’s wife also should be reported and followed.

### Recording and reporting of SAE

The investigator is responsible for the detection and documentation of events meeting the criteria and definition of a serious adverse event (SAE) as provided in this protocol. Each patient will be instructed to contact the investigator immediately should the subject manifest any signs or symptoms they perceive as serious.

SAEs will be reported concurrently to CRO, SPONSOR, Principle Investigator, IRB, and SFDA within 24 hours, once the investigator determines that the event meets the protocol definition of a SAE. The SAE Form will always be completed as thoroughly as possible with all available details of the event, signed by the investigator (or designee), and will always provide an assessment of causality at the time of the initial report.

After the initial SAE report, the investigator is required to actively follow each subject and provide further information to the CRO, SPONSOR, Principle Investigator, IRB, and SFDA on the subject’s condition. Investigators will follow-up subjects with SAEs until the event has: resolved or subsided or stabilized or disappeared or the event is otherwise explained, or the subject is lost to follow-up.

### Important noticeable event

Important noticeable events is defined as any event which must be solved and recorded, such as treatment stopping, dose reducing, concomitant medication and withdrawal.

### Procedure for causal relationship assessment

The confirmation of SAE and its causal relationship with the vaccines will be conducted by corresponding investigator and clinical monitor, and then reported to Principle investigator, CRO and SPONSOR. If the agreement for a certain SAE cannot be reached among investigator, CRO and SPONSOR, Adverse Event Evaluation Committee authorized by the Ministry of Health will therefore be invited for confirmation.

## Efficacy measurement

The efficacy measurement will rely on the following laboratory testing:

- 1. HBV DNA
  2. HBeAg
  3. HBeAb

### Definition of endpoints

The virologic response will be assessed four weeks after the end of treatment (week 24) and at the end of follow-up (week 44). HBeAg seroconversion was defined by the loss of HBeAg and the presence of anti-HBe antibody. Suppression of HBV DNA was defined as > 2 log10 decrease of viral load.

### Primary endpoint

The primary endpoint was defined as loss of HBeAg, or presence of anti-HBe antibody or suppression of HBV DNA.

### Secondary endpoint

The secondary endpoint was designated as both HBeAg seroconversion and suppression of HBV DNA.

- 1. Basic treatment

Considering the ethical issue, each patient who participates in this trial will be given basic treatment besides of study agents throughout 44-week study period as following:

- 1. Silymarin, 50 mg/tablets, 3 tablets per time, tid；
  2. Hu Gan Tablet, 0.36g per tablet, 4 tablets per time, tid.
  3. Concomitant medication

Concomitant medication is defined as: any vaccine, any other medication relevant to the protocol and administrated during the period starting from prior to treatment until the end of follow-up.

At each study center, the investigator should question the patient about any medication taken between two injections. All concomitant medication must be recorded in the CRFs with the trade name, generic name, indication, dose, route of administration, start and end dates of treatment.

The flowing drugs, treatments and behaviours are not allowed:

- 1. Any immunosuppressive or immunostimulant therapy, anti-viral therapy, drugs that have effects on decreasing the level of ALT, androgenic drugs, steroids, contraceptive drugs;
  2. Any investigational or non-registered anti-HBV drug, herbs or other patient self-chozen therapies
  3. Usage of acetaminophen more than 4 g per day;
  4. Intake of spirits more than 20 g per day;

1. Data management

## The System

A generic database management system (DBMS) based on EpiData has been designed by CRO. The system includes data entry, editing, reporting of errors and status of work, data documentation, security, and confidentiality of the data. Appropriate use of the system will ensure valid data are stored, complete and accurate, and that the necessary information is available for data analysis. The management control of the DBMS proposes the data are properly archived and stored at TigerMed (CRO), and enough precautionary measures are taken from accidental damage or loss of data. The design and management of the DBMS also promises confidentiality and non-violation of the human participation protection right.

## Data checking and coding

CRFs will be completed for each enrolled patient by investigators. After checking by clinical monitor, the original copy will be sent to data manager for data entry and data management. The in-house variables coding will be processed by data manager at TigerMed.

## Data checking, data entry and error checking

Batch processing system, which will benefit internal management of the data, such as data entry, error checking, updating, and reporting, has been chosen for the data entry get a sequential number. The data will be entered twice, and the key punching errors are to be detected by a module included in the system. The data errors are classified as incompleteness of the forms, duplications, range errors, inconsistencies, and link problems. Al these checks are included in a module within the system. Invoking the module will yield the list of all those kinds of problems in the data. These lists are to be used to resolve the problem and are to be kept properly as future reference. The detailed documentation of the check plans goes along with the system. The data errors will be reviewed and revised by investigator and clinical monitor before any modification with data entry system.

## Data freezing

Dataset will be modified in terms of the revisions targeted to error list from investigator. Any dataset modification and updating will be well documented for potential SFDA inspection in the future. After modification, the dataset will be frozen and transported to biostatistician for analysis.

# Statistical Methods

All data will be double entered into custom-made data entry programs. The data management programs include range and consistency checks. An SAS program (SAS Institute Inc., Cary, NC, USA ) will be applied for statistical analysis.

- 1. Safety

The safety analysis will be performed based on the safety set (SS). It will include patients who received at least one injection, and completed at least one interview of safety evaluation after injection.

Three-wise comparison employed the chi square test, or Fisher’s exact test when data were sparse, for dichotomous outcomes will be performed to test the difference of occurrence of adverse events among placebo, 30 μg YIC and 60 μg YIC groups. A p-value < 0.05 (two-tailed) will be considered statistically significant.

- 1. Efficacy

The efficacy analysis will be conducted based on both full analysis and per-protocol sets. For full analysis set (FAS), it will include patients who were randomized, received at least one injection, and completed at least one interview of efficacy evaluation after injection. The intent-to-treat principle will be applied for the dataset. For per protocol set (PPS), it will include patients who met following three conditions：

1. Baseline information are available；
2. Complete full course of treatment and follow-up without protocol violation；
3. With good compliance (between 80% and 120%）.

For those important indicators, the missing data will be estimated using the most previous observation (LOCF, last observation carry forwards).

The primary efficacy hypotheses will be tested for response rate of primary endpoint in 30 μg YIC or/and 60 μg YIC groups > that in placebo group (2-tailed). The Chi square test, or Fisher’s exact test when data were sparse, will be employed. A p-value, point estimate, as well as confidence interval will be calculated.

The secondary efficacy outcomes will be summarized in a similar fashion.

Repeated measures analysis will be performed using a generalized estimating equations (GEEs) method to adjust the dependence among repeated observations made on the same patient while testing the group and time effects. A p-value < 0.05 (two-tailed) will be considered statistically significant.

- 1. Sample size

The sample size was calculated to ensure an adequate evaluation of the primary endpoint. Based on the literatures and the results of phase IIa trial, a sample size of 78 patients per arm could detect a difference of primary response rate between 60 µg group, 30 µg group and placebo group (response rate in 60 µg YIC group vs placebo group, 35% vs 3%; and response rate in 30 µg YIC group vs placebo group, 20% vs 3%) with a statistical power of 80% at the 0.05 level of significance, allowing for a dropout rate of 20%.

# Coding and randomization

All enrolled patients will have two codes, one is screening code, another is randomization code (study agent code). The screening code will be assigned to each potential patient during the screening. It consists of 6 digitals. The first 3 digitals stands for treatment center, the second 3 digitals is serial number assigned to subjects in terms of the screening sequence by each center.

The randomization code will be generated by an independent biostatistician who will not be involved in this study, using SAS program (SAS 9.0). Randomization will occur separately at each study center. Block randomization method will be applied in blocks of six. The block size and seed number will be sealed with the randomization list. The randomization code will be masked with 3 digitals serial number from 001 to 240. Each study agent vial will be labeled with a 3-digital number. The labeling of study agent will be processed by the personnel who will not be involved in this study according to the randomization list under the supervision of SFDA at Beijing Vaccine Institute. A randomization list will be kept at SFDA.

After screening, the eligible patient will be given a study agent in terms of the sequence of recruitment.

# Packaging and labeling

- 1. Packaging

Study agent vial will be packaged in paper box. One box will contain one vial. Every 6 boxes will be collected in one package.

- 1. Labeling

Each study agent vial will be coded with a 3-digital randomization number, which is between 001 and 240. Besides, injection date, dosage, and lot number will be printed with randomization code. Additional vaccination precautions will be printed on the package label.

# Handling and distribution of study agent

CRO (TigerMed Ltd.) will provide and update the administration record form for each study center. Each enrolled patient will have a unique allocation number. The administration record form will record status of dose received and returned, and injection date.

Investigational clinical supplies (including study agents and basic drugs) must be received by a designated person at the study center, handled and stored safely and properly, and kept in a secured location to which only the investigator and designated assistants have access. Clinical supplies are to be administrated only in accordance with the protocol. The investigator is responsible for keeping accurate records of the clinical supplies received from the CRO, the amount administrated to the patients, and the amount remaining at the end of each injection. All investigational clinical supplies-related records must be provided to monitor for inspection at each schedule and unscheduled monitoring visit. Used (empty ampoule and package) and unused study agent and basic drug should be returned to SPONSOR via CRO at the end of study.

1. ETHICAL CONSIDERATIONS
   1. Protocol Review

Before initiation of the trial, the working protocol requires clearance by the Ethic Committee and Institutional Review Board (IRB) in Beijing Ditan Hospital, which is the principle investigation institute.

According to the international standard guidelines on IRB, an annual review will take place to evaluate if the research has been conducted according to the protocol which was approved by initial review. Investigators will submit a progress report, which includes: report of adverse events occurred, subject recruitment status etc.

- 1. Ethical Guidelines

The principles that govern biomedical research involving human subjects are of application to this project. The Declaration of Helsinki (appendix 2) and the International Conference on Harmonization’s Good Clinical Practice Guidelines (ICH-GCP ) (appendix 3) will be followed, aiming to provide assurance that the rights, integrity, and confidentiality of trial subjects are protected and that results reported are credible and accurate.

- 1. Informed Consent

Consents will be sought during the screening stage. All efforts will be put in place to so that consent obtention is: informed, given voluntarily and given by a competent person. Inform consent procedures will follow the WHO/TDR Guidelines.

- 1. Confidentiality

Participant confidentiality in publications, reports and clinical or biological specimens collected during the conduct of the trial and following completion of the trial, will be ensured. Clinical or biological specimens will have an ID number or laboratory number only. The list linking names of the participants to the ID or laboratory number will be kept separately. Access to both electronic and hard copy data will be restricted to authorized senior study personnel only.

- 1. Potential Risks and Risk Minimization

### Vaccination

The most common adverse event in phase IIa trial was local reaction at injection site, followed by arthralgia, and erythra. Almost all adverse events were mild. Participant who received YIC might appear temporary ALT elevation, which could be well treated by medication. Serious adverse event was not observed. Adverse events, if deemed related to the vaccination process will be treated at the treatment center without charge.

### Blood collection

The potential risk to the participants will be minimal, since all clinical procedures (venous blood collection) will be performed by adequately trained and experienced personnel under regular supervision. There is a small risk associated with phlebotomy for patients who are required to give a blood sample. This may include pain, redness and, very rarely, local infection at the phlebotomy area.

- 1. Protocol Modification

Any amendment to the trial, as the trial progresses, must be discussed by the investigator and SPONSOR concurrently. If agreement is reached concerning the need for an amendment, such amendment will be produced in writing and be made a formal part of the protocol. Any amendment requires Ethics Committee approval.

- 1. Stipends for Participation

Study participants will not receive stipends for participation in this trial.

- 1. Compensation

If a study participant develops a vaccine-related serious adverse event as confirmed by project principle investigator (Ditan Hospital), medical treatment will be provided according to local treatment guidelines and cost of such treatment will be charged to SPONSOR.

After completion of the study, patients who are assigned to placebo group will be given 6 months adefovir (anti-viral drug) treatment.

1. Compliance, monitoring, and archiving

By signing this protocol, the investigators agree to conduct the study in an efficient and diligent manner and in conformance with the protocol, generally accepted standards of GCP, and all requirements of SFDA regulations.

The investigators also agree to allow monitoring, audits, institutional Review Board/Independence Ethics Committee review, and regulatory agency inspection of trial-related documents and procedures and provide for direct access to all study-related source data and documents.

The investigators shall prepare and maintain complete and accurate study documentation in compliance with GCP standards and SFDA regulations, and for each patients participating in the study, provide all data, and upon completion or termination of the clinical study submit any other reports to the CRO and SPONSOR as required by this protocol or as otherwise required pursuant to any agreement with the CRO and SPONSOR. The investigators and institution must keep all trial documents for 5 years after licensure according to SFDA guideline.

Study document will be promptly and fully disclosed to the CRO or SPONSOR by the investigators upon request and also shall be made available at the investigators’ site upon request for inspection, coping, review, and audit at reasonable times by representatives of the CRO and SPONSOR, or any regulatory agencies. The investigators agree to promptly take any reasonable step that is requested by the CRO and SPONSOR as a result of an audit to cure deficiencies in the study documentation and worksheets/case report forms.

CRO and SPONSOR will ensure that the trial is adequately monitored. At regular intervals during the study, the study site will be contacted, through site visits, letters or telephone calls, by monitors appointed by CRO to review study progress, investigator and subject adherence to protocol requirements and any emergent problems. During monitoring visits, the following points will be scrutinized with the Investigator: subject informed consent, subject recruitment and follow-up, vaccine allocation, subject compliance to the follow up visits, adverse event documentation and reporting, data collection and quality, clinical specimens and product management and cold chain monitoring. A written report after each trial-site visit will be submitted to Tigermed and SPONSOR. The monitors will discuss any problem with the investigator and define, after conversation with SPONSOR before the actions to be taken.

1. PUBLICATION POLICY

All unpublished information, which relate to the therapeutic hepatitis B vaccine (YIC), such as patent application, product processing, formula for compounding, are confidential information.

Beijing Vaccine Institute and Shanghai Medical College, Fudan University jointly own the rights to all these confidential information, as well as the data, clinical and biological specimens, results and other findings resulting from this trial. The investigators shall insure not to disclose any confidential information without written authorization from both the Beijing Vaccine Institute and Shanghai Medical College, Fudan University. The trial results or findings will be published after trial closing and data analysis. Authors will include: recipient principal investigators, participating scientists and individuals who provide a substantial and credible contribution to the trial activities, including but not limited to: conduct of the study, data collection during the study, data analysis and/or report writing related to the study. Notwithstanding the foregoing, with the prior written approval of the Beijing Vaccine Institute and the Shanghai Medical College, Fudan University, the recipient may publish the results of the recipient’s work carried out in the study with the proper consideration of confidential issues. Generally, the Recipient shall provide the Beijing Vaccine Institute and the Shanghai Medical College a copy of the manuscript, abstract, or presentation at least 60 days before publication. To protect the patent and other rights, the Beijing Vaccine Institute and the Shanghai Medical College, Fudan University may ask recipient to postpone the publication for as least 60 days. Guidelines for authorship of major, international, peer-reviewed journals will be used to establish authorship. Recipient shall ensure that all SPONSORs that provide direct or indirect financial support or other resources for conduct of this study are acknowledged in such publications.

The Beijing Vaccine Institute and the Shanghai Medical College, Fudan University shall have the first right to publish the combined multi-center results of the study in abstract, report and/or peer-review scientific manuscript form. The principal investigators shall be included as co-authors and the contribution of the institutions involved will be acknowledged in all abstracts, reports, or other peer-reviewed scientific publications containing data or information collected during conduct of the study.

1. References

1. Beasley RP, Hwang LY, Lin CC, Chen CS, 1981. Hepatocellular carcinoma and hepatitis B virus: A prospective study of 22 707 men in Taiwan. . Lancet 2: 1129-33.

2. Wen YM, Qu D, Zhou SH, 1999. Antigen-antibody complex as therapeutic vaccine for viral hepatitis B. Int Rev Immunol 19: 251-8.

3. Wen YM, Wu XH, Hu DC, Zhang QP, Guo SQ, 1995. Hepatitis B vaccine and anti-HBs complex as approach for vaccine therapy. Lancet 345: 1575-6.

4. Xu DZ, Huang KL, Zhao K, Xu LF, Shi N, Yuan ZH, Wen YM, 2005. Vaccination with recombinant HBsAg-HBIG complex in healthy adults. Vaccine 23: 2658-64.

5. Yao X, Zheng BJ, Zhou J, Xu DZ, Zhao K, Sun SH, Yuan ZH, Wen YM, 2007. Therapeutic effect of hepatitis B surface antigen-antibody complex is associated with cytolytic and non-cytolytic immune responses in hepatitis B patients. Vaccine 25: 1771-9.

6. Zheng BJ, Ng MH, He LF, Yao X, Chan KW, Yuen KY, Wen YM, 2001. Therapeutic efficacy of hepatitis B surface antigen-antibodies-recombinant DNA composite in HBsAg transgenic mice. Vaccine 19: 4219-25.

Appendix 1. List of abbreviations

| AE | Adverse event |
| --- | --- |
| AFP | Alpha fetoprotein |
| ALB | Albumin BCG |
| ALT | Alanine aminotransferase |
| Anti-HBc | Antibody to Hepatitis B Core Antigen |
| Anti-HBe | Antibody to Hepatitis Be Antigen |
| Anti-HBs | Antibody to Hepatitis B Surface Antigen |
| ANOVA | Analysis of variance |
| AST | Aspartate aminotransferase |
| BUN | Blood urea nitrogen |
| CMH | Cochran-Mantel-Haenszel |
| Cr | Creatinine |
| CRF | Case report form |
| CTL | Cytotoxic T lymphocytes |
| DBIL | Direct Bilirubin |
| DRQ | Data Ramp query |
| ECG | Electrocardiogram |
| GCP | Good Clinical Practice |
| HAV | Hepatitis A Virus |
| HBIG | Hepatitis B immunoglobulin |
| HBsAg | Hepatitis B Surface Antigen |
| HBV DNA | Hepatitis B virus DNA |
| HCG | Human chorionic gonadotropin |
| HCV | Hepatitis C virus |
| HDL | High density lipoprotein |
| HDV | Hepatitis D virus |
| HEV | Hepatitis E virus |
| HIV | Human immunodeficiency virus |
| IC | Immune Complex |
| ICF | Informed consent forms |
| ICH | International Conference on Harmonization |
| IEC | Independent Ethics Committee |
| IRB | Institutional Review Board |
| ITT | Intent to treat |
| LDL | Low density lipoprotein |
| LOCF | Last Observation Carry Forward |
| pH | Hydrogen ion concentration |
| PP | “Per-protocol” population |
| PT | Prothrombin Time |
| PTA | Plasma thromboblastin antecedent |
| SAE | Serious adverse event |
| SFDA | China’s State Food and Drug Administration |
| TP | Total Protein |
| TBIL | Total Bilirubin |
| YMDD | tyrosine-methionine-aspartate-aspartate |

appendix 2. Flow chart

| **Proceduree** | **Screening**  **（Day）** | **baseline** | **Treatment（Week±4days）** | | | | | | **Follow-up （week±4days）** | |
| --- | --- | --- | --- | --- | --- | --- | --- | --- | --- | --- |
|  | **-14 to ‑1** | **0** | **4** | **8** | **12** | **16** | **20** | **24** | **36** | **44** |
| Inform consent | **×** |  |  |  |  |  |  |  |  |  |
| Recruitment criteria evaluation |  | **×** |  |  |  |  |  |  |  |  |
| Study agent injection |  | **×** | **×** | **×** | **×** | **×** | **×** |  |  |  |
| Medical history investigation | **×** |  |  |  |  |  |  |  |  |  |
| Physical examination（Temp, heart rate, and blood presure） | **×** |  | **×** | **×** | **×** | **×** | **×** | **×** | **×** | **×** |
| X-ray (a)、type B ultrasonic (liver, spleen and gallbladder) (c) | **×** |  |  |  |  |  |  |  |  |  |
| electrocardiogram | **×** |  |  |  |  |  |  | **×** |  |  |
| Urine or blood HCG pregnancy test (b) | **×** |  |  |  |  |  |  |  |  |  |
| HBV virologic testing | **×** |  |  |  |  |  |  |  |  |  |
| HBV-DNAquantity analysis | **×** |  |  |  |  |  |  |  |  |  |
| Liver function （ALT、AST、TBil、DBil、TP、ALB、AKP），renal function (BUN、Cr)， K， Na，Cl，blood glocuse* | **×** |  | **×** | **×** | **×** | **×** | **×** | **×** | **×** | **×** |
| alpha fetoprotein（AFP） | **×** |  |  |  |  |  |  | **（X）** |  |  |
| Anti-HBc | **×** |  |  |  |  |  |  | **×** |  |  |
| Blood cell count (d)，PTA，urine test(d) | **×** |  | **×** | **×** | **×** | **×** | **×** | **×** | **×** | **×** |
| Anti-HAV IgM, anti-HDV, anti-HEV, anti-HCV, anti-HIV | **×** |  |  |  |  |  |  |  |  |  |
| Serum sampling for virologic response testing | **×** |  |  |  | **×** |  |  | **×** | **×** | **×** |
| Basic treatment |  | **×** | **×** | **×** | **×** | **×** | **×** | **×** | **×** | **×** |
| Record concomitant therapy | **×** | **×** | **×** | **×** | **×** | **×** | **×** | **×** | **×** | **×** |
| Adverse event investigation | **×** | **×** | **×** | **×** | **×** | **×** | **×** | **×** | **×** | **×** |
| Diary card fill in out by patients |  | **×** | **×** | **×** | **×** | **×** | **×** | **×** | **×** | **×** |
| 1. **Patients with** pulmonary disease. Except for those patients stay at stable phase and w**ith chest x-ray diagnosis in the preceding 12 months.** 2. **Productive age women will be tested 24 hours prior to treatment.** 3. **Patients with hepatic cirrhosis (including suspected hepatic cirrhosis) or AFP abnormal should have imageologic diagnosis before recruitment to exclude possibility of liver tumour.** 4. **Patients should be followed-up regularly till recovery or reaching the baseline level, if there is any abnormality of laboratory tests. Uric haematoglobin positive should be confirmed with reagent paper and microscopy.** | | | | | | | | | | |

Appendix 3. WORLD MEDICAL ASSOCIATION DECLARATION OF HELSINKI

Adopted by the 18th WMA General Assembly, Helsinki, Finland, June 1964, and amended by the
29th WMA General Assembly, Tokyo, Japan, October 1975
35th WMA General Assembly, Venice, Italy, October 1983
41st WMA General Assembly, Hong Kong, September 1989
48th WMA General Assembly, Somerset West, Republic of South Africa, October 1996
and the 52nd WMA General Assembly, Edinburgh, Scotland, October 2000
Note of Clarification on Paragraph 29 added by the WMA General Assembly, Washington 2002
Note of Clarification on Paragraph 30 added by the WMA General Assembly, Tokyo 2004

1. INTRODUCTION
   1. The World Medical Association has developed the Declaration of Helsinki as a statement of ethical principles to provide guidance to physicians and other participants in medical research involving human subjects. Medical research involving human subjects includes research on identifiable human material or identifiable data.
   2. It is the duty of the physician to promote and safeguard the health of the people. The physician's knowledge and conscience are dedicated to the fulfillment of this duty.
   3. The Declaration of Geneva of the World Medical Association binds the physician with the words, "The health of my patient will be my first consideration," and the International Code of Medical Ethics declares that, "A physician shall act only in the patient's interest when providing medical care which might have the effect of weakening the physical and mental condition of the patient."
   4. Medical progress is based on research which ultimately must rest in part on experimentation involving human subjects.
   5. In medical research on human subjects, considerations related to the well-being of the human subject should take precedence over the interests of science and society.
   6. The primary purpose of medical research involving human subjects is to improve prophylactic, diagnostic and therapeutic procedures and the understanding of the aetiology and pathogenesis of disease. Even the best proven prophylactic, diagnostic, and therapeutic methods must continuously be challenged through research for their effectiveness, efficiency, accessibility and quality.
   7. In current medical practice and in medical research, most prophylactic, diagnostic and therapeutic procedures involve risks and burdens.
   8. Medical research is subject to ethical standards that promote respect for all human beings and protect their health and rights. Some research populations are vulnerable and need special protection. The particular needs of the economically and medically disadvantaged must be recognized. Special attention is also required for those who cannot give or refuse consent for themselves, for those who may be subject to giving consent under duress, for those who will not benefit personally from the research and for those for whom the research is combined with care.
   9. Research Investigators should be aware of the ethical, legal and regulatory requirements for research on human subjects in their own countries as well as applicable international requirements. No national ethical, legal or regulatory requirement should be allowed to reduce or eliminate any of the protections for human subjects set forth in this Declaration.
2. BASIC PRINCIPLES FOR ALL MEDICAL RESEARCH
   1. It is the duty of the physician in medical research to protect the life, health, privacy, and dignity of the human subject.
   2. Medical research involving human subjects must conform to generally accepted scientific principles, be based on a thorough knowledge of the scientific literature, other relevant sources of information, and on adequate laboratory and, where appropriate, animal experimentation.
   3. Appropriate caution must be exercised in the conduct of research which may affect the environment, and the welfare of animals used for research must be respected.
   4. The design and performance of each experimental procedure involving human subjects should be clearly formulated in an experimental protocol. This protocol should be submitted for consideration, comment, guidance, and where appropriate, approval to a specially appointed ethical review committee, which must be independent of the investigator, the SPONSOR or any other kind of undue influence. This independent committee should be in conformity with the laws and regulations of the country in which the research experiment is performed. The committee has the right to monitor ongoing trials. The researcher has the obligation to provide monitoring information to the committee, especially any serious adverse events. The researcher should also submit to the committee, for review, information regarding funding, SPONSORs, institutional affiliations, other potential conflicts of interest and incentives for subjects.
   5. The research protocol should always contain a statement of the ethical considerations involved and should indicate that there is compliance with the principles enunciated in this Declaration.
   6. Medical research involving human subjects should be conducted only by scientifically qualified persons and under the supervision of a clinically competent medical person. The responsibility for the human subject must always rest with a medically qualified person and never rest on the subject of the research, even though the subject has given consent.
   7. Every medical research project involving human subjects should be preceded by careful assessment of predictable risks and burdens in comparison with foreseeable benefits to the subject or to others. This does not preclude the participation of healthy volunteers in medical research. The design of all studies should be publicly available.
   8. Physicians should abstain from engaging in research projects involving human subjects unless they are confident that the risks involved have been adequately assessed and can be satisfactorily managed. Physicians should cease any investigation if the risks are found to outweigh the potential benefits or if there is conclusive proof of positive and beneficial results.
   9. Medical research involving human subjects should only be conducted if the importance of the objective outweighs the inherent risks and burdens to the subject. This is especially important when the human subjects are healthy volunteers.
   10. Medical research is only justified if there is a reasonable likelihood that the populations in which the research is carried out stand to benefit from the results of the research.
   11. The subjects must be volunteers and informed participants in the research project.
   12. The right of research subjects to safeguard their integrity must always be respected. Every precaution should be taken to respect the privacy of the subject, the confidentiality of the patient's information and to minimize the impact of the study on the subject's physical and mental integrity and on the personality of the subject.
   13. In any research on human beings, each potential subject must be adequately informed of the aims, methods, sources of funding, any possible conflicts of interest, institutional affiliations of the researcher, the anticipated benefits and potential risks of the study and the discomfort it may entail. The subject should be informed of the right to abstain from participation in the study or to withdraw consent to participate at any time without reprisal. After ensuring that the subject has understood the information, the physician should then obtain the subject's freely-given informed consent, preferably in writing. If the consent cannot be obtained in writing, the non-written consent must be formally documented and witnessed.
   14. When obtaining informed consent for the research project the physician should be particularly cautious if the subject is in a dependent relationship with the physician or may consent under duress. In that case the informed consent should be obtained by a well-informed physician who is not engaged in the investigation and who is completely independent of this relationship.
   15. For a research subject who is legally incompetent, physically or mentally incapable of giving consent or is a legally incompetent minor, the investigator must obtain informed consent from the legally authorized representative in accordance with applicable law. These groups should not be included in research unless the research is necessary to promote the health of the population represented and this research cannot instead be performed on legally competent persons.
   16. When a subject deemed legally incompetent, such as a minor child, is able to give assent to decisions about participation in research, the investigator must obtain that assent in addition to the consent of the legally authorized representative.
   17. Research on individuals from whom it is not possible to obtain consent, including proxy or advance consent, should be done only if the physical/mental condition that prevents obtaining informed consent is a necessary characteristic of the research population. The specific reasons for involving research subjects with a condition that renders them unable to give informed consent should be stated in the experimental protocol for consideration and approval of the review committee. The protocol should state that consent to remain in the research should be obtained as soon as possible from the individual or a legally authorized surrogate.
   18. Both authors and publishers have ethical obligations. In publication of the results of research, the investigators are obliged to preserve the accuracy of the results. Negative as well as positive results should be published or otherwise publicly available. Sources of funding, institutional affiliations and any possible conflicts of interest should be declared in the publication. Reports of experimentation not in accordance with the principles laid down in this Declaration should not be accepted for publication.
3. ADDITIONAL PRINCIPLES FOR MEDICAL RESEARCH COMBINED WITH MEDICAL CARE
   1. The physician may combine medical research with medical care, only to the extent that the research is justified by its potential prophylactic, diagnostic or therapeutic value. When medical research is combined with medical care, additional standards apply to protect the patients who are research subjects.
   2. The benefits, risks, burdens and effectiveness of a new method should be tested against those of the best current prophylactic, diagnostic, and therapeutic methods. This does not exclude the use of placebo, or no treatment, in studies where no proven prophylactic, diagnostic or therapeutic method exists. [See footnote](http://www.wma.net/e/policy/b3.htm#note1%23note1)
   3. At the conclusion of the study, every patient entered into the study should be assured of access to the best proven prophylactic, diagnostic and therapeutic methods identified by the study. [See footnote](http://www.wma.net/e/policy/b3.htm#note2%23note2)
   4. The physician should fully inform the patient which aspects of the care are related to the research. The refusal of a patient to participate in a study must never interfere with the patient-physician relationship.
   5. In the treatment of a patient, where proven prophylactic, diagnostic and therapeutic methods do not exist or have been ineffective, the physician, with informed consent from the patient, must be free to use unproven or new prophylactic, diagnostic and therapeutic measures, if in the physician's judgement it offers hope of saving life, re-establishing health or alleviating suffering. Where possible, these measures should be made the object of research, designed to evaluate their safety and efficacy. In all cases, new information should be recorded and, where appropriate, published. The other relevant guidelines of this Declaration should be followed.

**Note**: **Note of clarification on paragraph 29 of the WMA Declaration of Helsinki**

The WMA hereby reaffirms its position that extreme care must be taken in making use of a placebo-controlled trial and that in general this methodology should only be used in the absence of existing proven therapy. However, a placebo-controlled trial may be ethically acceptable, even if proven therapy is available, under the following circumstances:

  - Where for compelling and scientifically sound methodological reasons its use is necessary to determine the efficacy or safety of a prophylactic, diagnostic or therapeutic method; or

  - Where a prophylactic, diagnostic or therapeutic method is being investigated for a minor condition and the patients who receive placebo will not be subject to any additional risk of serious or irreversible harm.

All other provisions of the Declaration of Helsinki must be adhered to, especially the need for appropriate ethical and scientific review.

[Page back to paragraph 29](http://www.wma.net/e/policy/b3.htm#paragraphe29%23paragraphe29).

**Note**: **Note of clarification on paragraph 30 of the WMA Declaration of Helsinki**

The WMA hereby reaffirms its position that it is necessary during the study planning process to identify post-trial access by study participants to prophylactic, diagnostic and therapeutic procedures identified as beneficial in the study or access to other appropriate care. Post-trial access arrangements or other care must be described in the study protocol so the ethical review committee may consider such arrangements during its review.

[Page back to paragraph 30](http://www.wma.net/e/policy/b3.htm#paragraphe30%23paragraphe30).

The Declaration of Helsinki (Document 17.C) is an official policy document of the World Medical Association, the global representative body for physicians. It was first adopted in 1964 (Helsinki, Finland) and revised in 1975 (Tokyo, Japan), 1983 (Venice, Italy), 1989 (Hong Kong), 1996 (Somerset-West, South Africa) and 2000 (Edinburgh, Scotland). Note of clarification on Paragraph 29 added by the WMA General Assembly, Washington 2002.

Appendix 4. THE PRINCIPLES OF ICH GCP

1. Clinical trials should be conducted in accordance with the ethical principles that have their origin in the Declaration of Helsinki, and that are consistent with GCP and the applicable regulatory requirement(s).
2. Before a trial is initiated, foreseeable risks and inconveniences should be weighed against the anticipated benefit for the individual trial subject and society. A trial should be initiated and continued only if the anticipated benefits justify the risks.
3. The rights, safety, and well being of the trial subjects are the most important considerations and should prevail over interests of science and society.
4. The available non-clinical and clinical information on an investigational product should be adequate to support the proposed clinical trial.
5. Clinical trials should be scientifically sound, and described in a clear, detailed protocol.
6. A trial should be conducted in compliance with the protocol that has received prior institutional review board (IRB)/independent ethics committee (IEC) approval/favorable opinion.
7. The medical care given to, and medical decisions made on behalf of, subjects should always be the responsibility of a qualified physician or, when appropriate, of a qualified dentist.
8. Each individual involved in conducting a trial should be qualified by education, training, and experience to perform his or her respective task(s).
9. Freely given informed consent should be obtained from every subject prior to clinical trial participation.
10. All clinical trial information should be recorded, handled, and stored in a way that allows its accurate reporting, interpretation and verification.
11. The confidentiality of records that could identify subjects should be protected, respecting the privacy and confidentiality rules in accordance with the applicable regulatory requirement(s).
12. Investigational products should be manufactured, handled, and stored in accordance with applicable good manufacturing practice (GMP). They should be used in accordance with the approved protocol.

1. [↑](#endnote-ref-2)
2. Only for patients with pulmonary disease. Except for those patients stay at stable phase and with chest x-ray diagnosis in the preceding 12 months. [↑](#footnote-ref-2)
3. Only for patients with hepatic cirrhosis (including suspected hepatic cirrhosis) or AFP abnormal should have imageologic diagnosis before recruitment to exclude possibility of liver tumour. [↑](#footnote-ref-3)
4. Productive age women will be tested 24 hours prior to treatment. [↑](#footnote-ref-4)
5. Patients will be followed-up regularly till recovery or reaching the baseline level, if there is any abnormality of laboratory tests. Uric haematoglobin positive should be confirmed with reagent paper and microscopy. [↑](#footnote-ref-5)
